# Historical descriptions of nystagmus and abnormal involuntary eye movements in various ancient cultures

**DOI:** 10.1177/00368504231191986

**Published:** 2023-08-29

**Authors:** Johannes Gerb, Thomas Brandt, Doreen Huppert

**Affiliations:** 1 Department of Neurology, 9183 Ludwig-Maximilians-Universität, Munich, Germany; 2 German Center for Vertigo and Balance Disorders-DSGZ, 9183 Ludwig-Maximilians-Universität, Munich, Germany; 3 Clinical Neuroscience, 9183 Ludwig-Maximilians-Universität, Munich, Germany

**Keywords:** Nystagmus, ocular oscillations, antiquity, historical observations, history of eye movements, neuro-ophthalmology

## Abstract

Original texts and expert translations from various ancient cultures covering a time span from the 2^nd^ millennium BC to the ninth century AD were searched for historical observations of involuntary eye movements. Abnormal, spontaneous eye movements are an easily recognisable neuro-ophthalmological symptom that can be both congenital and acquired. Ocular oscillations termed ‘hippos’ by Hippocrates (460–370BC) and Galenos (129–216AD) are synonymous with nystagmus, a term first introduced in the eighteenth century. The original description of hippos suggests an innate onset, which retrospectively can be related to either congenital (infantile) nystagmus or continuous involuntary eye movements of the blind. Other descriptions of abnormal involuntary eye movements with different beating directions, possibly associated with vertigo, seizures or ear symptoms and their impact on patients’ quality of life (e.g. oscillopsia, blurred vision) are preserved in many fragmentary ancient documents including papyrus scrolls and stone tablets from Egypt, Mesopotamia, India, China, Greece, Rome and the Middle East. Although the sparse original descriptions of the direction and type of eye movements may inspire daring medical interpretations, caution is required when attempting to assign them to distinct nystagmus forms according to our current clinical classification of ocular motor disorders.

## Introduction

Physiological nystagmus includes the vestibulo-ocular reflex (which keeps an image stable on the retina during head accelerations by means of the slow phase of the nystagmus) and the optokinetic reflex during exposure to moving visual patterns. Involuntary pathological ocular oscillations or nystagmus occur in a variety of vestibular, ocular or central nervous system disorders, in particular infratentorial brainstem and cerebellar dysfunctions.^1^ They may be episodic, as in Menière's disease or vestibular migraine, both of which were already described in Chinese and Greek antiquity.^2^ They may be chronic, as in congenital (infantile) nystagmus or in the form of irregular eye movements in the blind.^1^ As stated by Wade and Tatler^3^ in their book on the origins of eye movement research, there are numerous historical depictions of optic phenomena and diseases of the eyes, but only a few about eye movements.^4^ However, ancient observations of unwanted ocular oscillations can be expected, since nystagmus is a striking and frequent neurological sign with distressing symptoms. Patients complain about impairment of visual acuity, apparent motion of the visual scene (oscillopsia), dizziness or double vision in disconjugated binocular oscillations. It was the observation of Hippocrates of Kos (Ἱπποκράτης ὁ Κῷος, ca. 460BC – ca. 370BC),^5^ later stated in works attributed to Galenos of Pergamon (Γαληνός, ca. 130AD – between 199 and 216AD),^6^ which prompted us to search sources from several ancient cultures for other reports of pathological involuntary eye movements:

Hippos is a congenital condition in which the eyes are in a constant state of restless motion … Hippocrates named this condition hippos. It is a malady of the muscles which surround the base of the globe and hold the eye in its place […]^6^

## Methods

We searched original literature, translations and existing compilations of ancient medical texts for observations of involuntary ocular oscillations dated between the sixteenth century BC and the 9^th^ century AD. Historical sources relate to cultures in Egypt, Mesopotamia, India, Greece, Roman Empire, China and the Middle East. Search terms were broad (e.g. ‘eyes’, ‘eyeballs’ and their linguistic derivations) to include all texts on involuntary eye movements. Further terms included ‘spinning’, ‘drifting’, ‘trembling’ and ‘shaking’, that is, symptoms of eye movements associated with vertigo. English, French and German search terms were applied in translated literature, while Latin (vertigo, caligo, bulbus, oculus, tremor, spasmus) and Greek (σκοτοδινίη, ἴλλιγγος, ὀφθαλμός, νυσταγμός, νυστάζειν, σπασμός) words and their derivations were used in primary sources. Comprehensive ancient medical texts such as Huangdi Neijing^7^ and Sushrutas s*amhita-uttura-tantra*^8^ were analysed in the original form and employing different translations.

## Results

**Figure 1.**
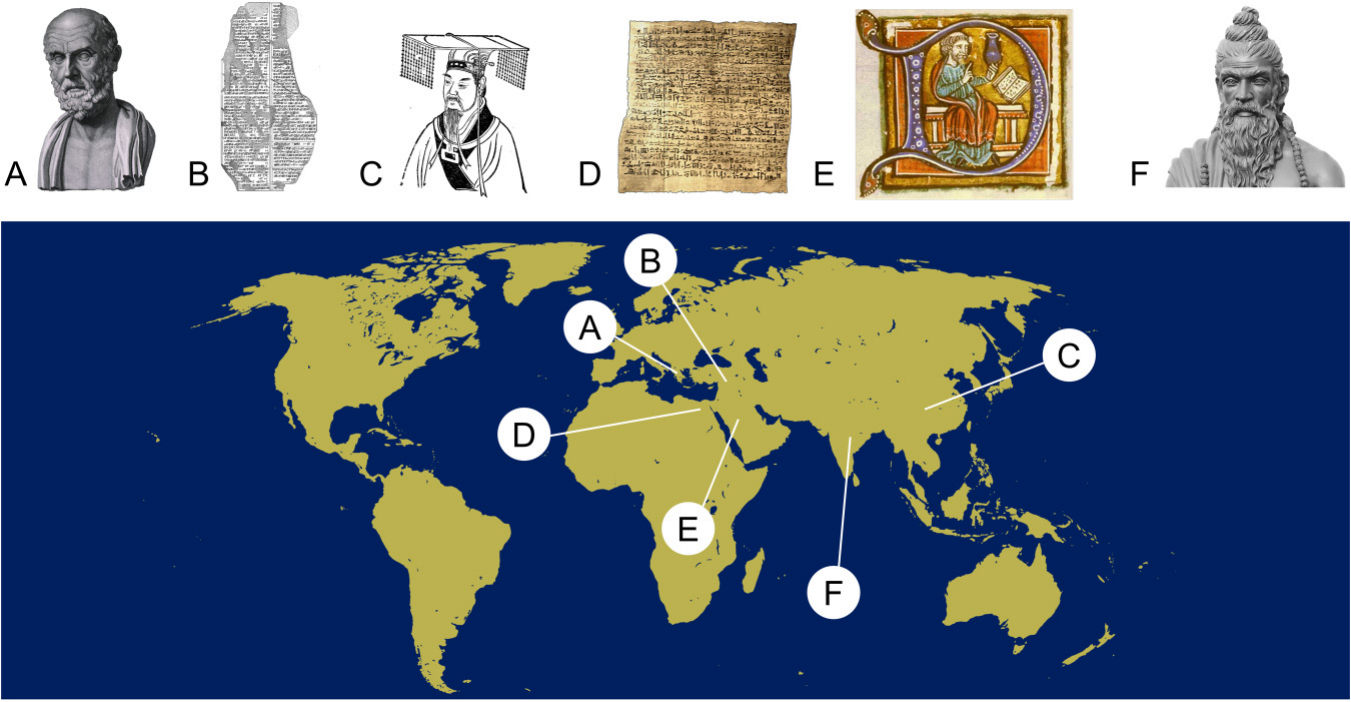
World map depicting the geographical origins of the aforementioned text segments. (a) Bust of Hippocrates of Kos who described ‘hippos’ as the rhythmic involuntary movement of the eyeballs. (b) Line art drawing of the Sultantepe tablets, dating back to ancient Mesopotamia. (c) Yellow emperor, a mystical figure of Chinese mythology whose conversations with his legendary ministers constitute the Huangdi Neijing, an early compendium of Chinese medicine which includes depictions of eye movements. (d) Edwin Smith Papyrus, an Egyptian collection of medical cases. (e) Drawing of Hunain ibn Is-hâq, the author of the first Arabian book on ophthalmology. (f) Head of statue depicting a rendition of Indian scholar Sushruta, to whom ophthalmological works are attributed (image sources: Wikimedia commons (A, C, E, F; respective modifications: crop, color correction), Cuneiform Digital Library Initiative (B), Breasted JH: ‘The Edwin Smith Papyrus’^9^ (D)).

We found likely descriptions of pathological eye movements in various ancient cultures (Figure 1) with different levels of detail and medical accuracy, examples of which are depicted chronologically (cp. Table 1). When available, ancient ideas of pathomechanisms and treatment options are provided.

**Table 1.** Example text segments containing descriptions of involuntary eye movements from ancient Egypt, Mesopotamia, India, China, Greece/Rome and the Middle East. Ancient and possible modern interpretations are provided.

	Text segment	Source	Ancient inter-pretation	Ancient treatment	Possible modern interpretation
Egypt	‘One having a gaping wound in his head, penetrating to the bone, perforating the sutures of his skull; he has developed ‘ty’, his mouth is bound, (and) he suffers with stiffness in his neck. […] His mouth is bound, (and) both his eye (brows) are drawn/shaking/distorted, while his face is as if he wept […]’	Edwin Smith Papyrus, ca. 1650–1550 BC	Not available	Warm dressings for neck stiffness	Possible case of neuroinfection with associated (vertical) nystagmus
Mesopotamia	‘If/when it comes over him, his right eye makes (a bobbing motion) like a spindle whorl (when) it [spins] (and) his left eye is full of blood, [he continually opens] his mouth, (and) he bites [his tongue], LUGAL.GÌR.RA afflicts him […] continually pursues him […]’	Sultantepe Tablets; before 6^th^ century BC	Demonic possession	Rituals/ ceremonies	Possible depiction of epileptic seizures with (pendular) nystagmus
India	‘In a case of Sannipatika Timira, the outer world looks variegated and confused, appears as doubled or trebled to the vision (of the patient), and stars and planets, either defective or supplied with additional limbs, seem to float about in the vision.’	Sushruta Samhita (सुश्रुतसंहिता); 2^nd^ to 1^st^ century BC	Not available	Not available; modern-day ayurvedic medicine: herbal remedies	Nystagmic oscillopsia
China	‘In severe cases, [patients hear] a ringing [sound] in the ears and [they experience] dizziness and vertigo. Their eyes fail to recognize other persons. They tend to suddenly fall.’	Huangdi Neijing (黄帝内经); 1^st^ to 8^th^ century AD	Qi imbalance	Re-balancing of Qi, e.g. dietary measures	Possible peripheral-vestibular nystagmus during an attack of Menière's Disease
Greece/Rome	[…] This type of headache is called heterocrania…The face is distorted spasmodically, the eyes remain glassy and rigid like horns or move to and fro forcedly, and the patient is dizzy […]	‘De causis et signis acutorum morborum’ by Aretaios of Cappadocia (Ἀρεταῖος, 80/81 AD – ca. 130-138 AD)	Not available	Not available	Possible depiction of central nystagmus in vestibular migraine
Greece/Rome	‘It happens then sometimes in the case of one eye, sometimes of both, from some blow, or from epilepsy, or from a spasm, by which the eyeball itself is violently shaken, that it cannot be directed at any object, or be held at all steady, but with no reason it turns now this way, now that, and so does not even afford a view of objects.’	‘De medicina’ by Aulus Cornelius Celsus (ca. 25 BC - ca. 50 AD)	Epilepsy, trauma	Not available	Different possible etiologies (trauma, epilepsy) of nystagmic oscillopsia
Greece/Rome	[…] it is similar to an eye disease . . . which is called hippos, in which it is not possible to keep the eyes still for a moment. . . they are forever oscillating back and forth with a trembling movement […]	‘Definitiones medicae’, attributed to Galenos of Pergamon (Γαληνός, ca. 130AD – between 199 and 216AD)	Not available	Not available	Clarification of terminology
Middle East	The lesions happening to the voluntary motion of the eye are of three kinds: in the first the motion ceases; this is called paralysis, laming; in the second it is diminished, and this is called numbness and trembling; in the third the voluntary motion is a disturbed one, i.e. other than it is the intention of the moving agent to produce, and this is called spasm.	‘Liber de oculis’ by Hunain ibn Is-hâq (808–873 AD)	Not available	Not available	Possible description of ophthalmoplegia, (partial) ocular muscle paresis and nystagmus

### Egypt

**Figure 2.**
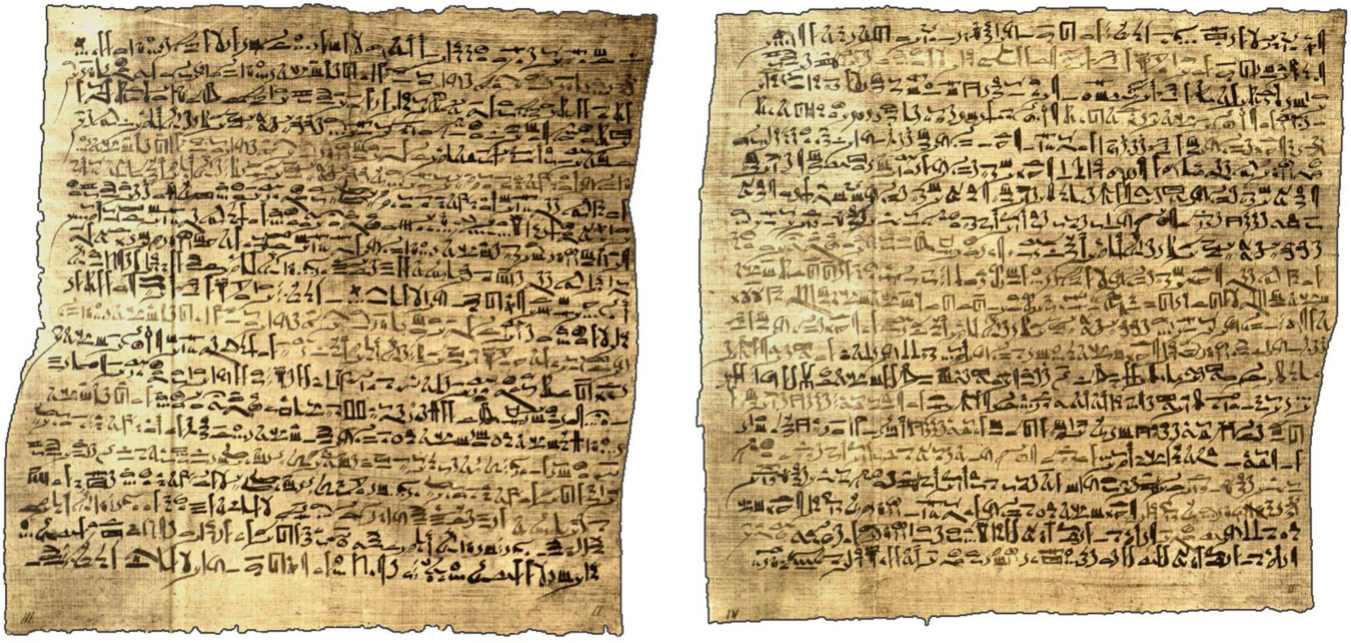
Excerpt from the Edwin Smith Papyrus.^9^ In this Egyptian collection of mostly surgical cases, case 7 describes the initial and follow-up examination of a patient with a penetrating skull trauma who developed fever, neck stiffness and ocular symptoms.

The Edwin Smith Papyrus (ca. 1650–1550 BC^9^; Figure 2) is a collection of 48 mostly surgical cases such as injuries, fractures, wounds, dislocations or tumours. The seventh case describes a patient suffering from head trauma with skull perforation presenting with ocular symptoms noted as ‘eyebrows being askew/shaking/distorted’ depending on the particular translations, e.g. ‘n ḥr.w’ (upwards), ‘n ẖr.w’ (downwards) and ‘ṯrm’ (to wink) or as a bobbing/twitching motion (literally ‘making a case of (drawing upwards), (and) a case of (drooping downwards).’).^9,10^ This could be interpreted as a vertical nystagmus.

‘One having a gaping wound in his head, penetrating to the bone, perforating the sutures of his skull; he has developed ‘ty’, his mouth is bound, (and) he suffers with stiffness in his neck. […] His mouth is bound, (and) both his eye (brows) are drawn/shaking/distorted, while his face is as if he wept […]’^9^

While neither treatment options for the wound nor the (possible) eye movements are provided, the neck stiffness is treated symptomatically by application of heat (e.g. warm dressings, comprised of grease, honey and lint).^9^

### Mesopotamia

**Figure 3.**
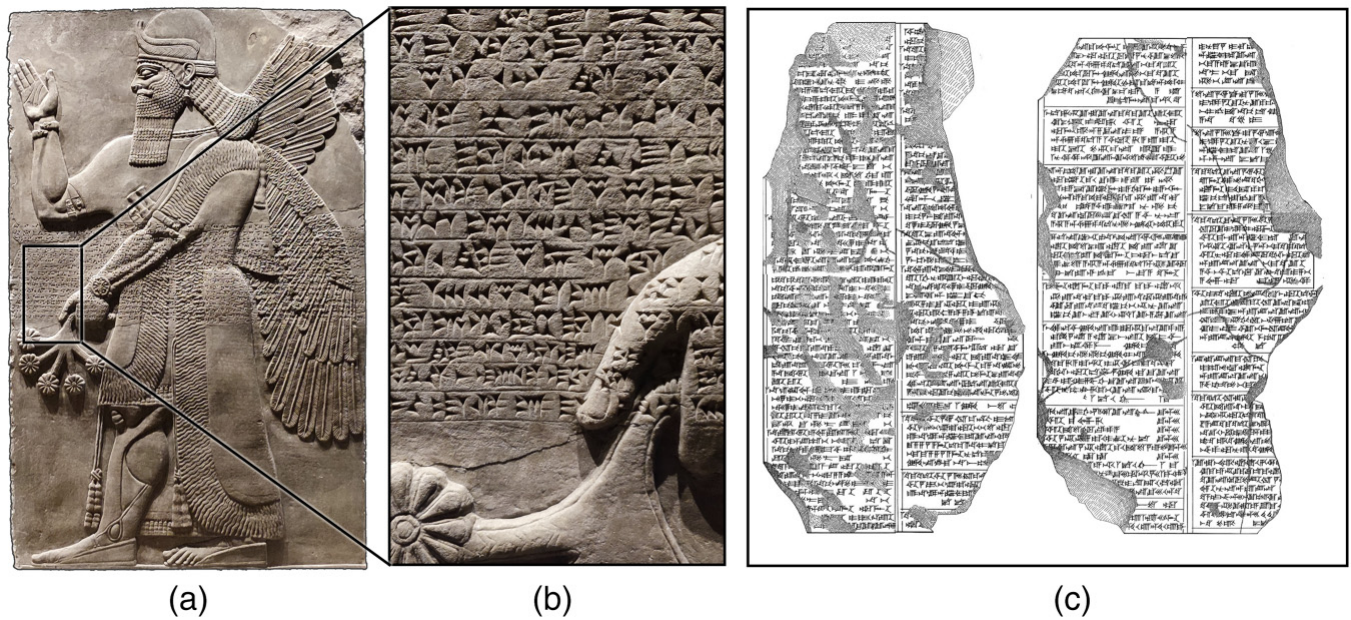
(a) Stone relief from the palace of Aššur-nâṣir-apli II, approx. 870 BC, showing a winged genie. (b) Detailed closeup of the embedded cuneiform script. It illustrates the sparse fragmentary information on which current meaningful interpretations are based (image sources a, b: own photograph). (c) Line art drawing of the *Sultantepe tablets* describing various ophthalmological symptoms with oscillatory character (initial publication by O. Gurney,^12^ digitised by the Cuneiform Digital Library Initiative^14^).

Cuneiform stone tablets (Figure 3) from the library of Aššur-bāni-apli, built during his reign from 668–627 BC in Nineveh (currently Iraq), and from the site of Sultantepe (currently Turkey) deal with disorders including those of eye movements.^11,12^ Scurlock and Andersen^13^ collected sections in which disease-related pathological eye movements are described as parādu (to shudder), arāru (to tremble), ṭamû (to twine like when spinning wool between one’s fingers), lamû (to spin like a spindle whorl), galātu (to jerk), neqelpû (to drift downstream) and ṣapāru (to move back and forth):

If/when it comes over him, his right eye makes (a bobbing motion) like a spindle whorl (when) it [spins] (and) his left eye is full of blood, [he continually opens] his mouth, (and) he bites [his tongue], LUGAL GÌRRA afflicts him [. . .]

If/when it comes over him, his right hand (and) the [left] side of his face [. . .] he growls like a dog, [he grinds?] his teeth, (and) his eyes jerk, LUGAL GÌRRA afflicts him, the āšipu should not make a prognostication as to his recovery.

The aforementioned Lugal Gìrra is a lower demon associated with Šulpaea, a second-rank god in the Mesopotamian pantheon, while āšipu refers to the treating physician. Scurlock and Andersen attribute these text segments to distinct nystagmus forms such as periodic alternating nystagmus, pendular nystagmus, vestibular nystagmus in Menière's disease or epileptic nystagmus due to cerebral malaria.^13^

Another depiction of hemicrania and vertical involuntary eye movements could be read as central vertical nystagmus in vestibular migraine.

If his right temple hurts him and (his eyes) drift downstream, [it is] ‘hand’ of Sîn.

Further hints on pathological eye movements were reported by Geller, who investigated numerous accounts of epilepsy in ancient Mesopotamia^15^ with seizures associated with abnormal eye movements:

If a sick man's neck turns to the right, time and again, while his hands and feet are paralysed (amšā), his eyes are now closed, now rolling, saliva flows in/from his mouth, he makes […] sound (harāru): [it is] A N TA ŠU B BA.

A n t a š u b b a, Sumerian for ‘what has fallen from heaven’, is a term solely used in scientific Babylonian literature referring to a distinct form of epileptic seizures, possibly characterised by the discharge of saliva. Due to the characterisation as ‘demonic’ afflictions, therapeutical interventions were mostly religious, for example, ceremonies or rituals.

### India

The Sushruta-Samhita^8^ is a compendium of medical texts attributed to the Indian scholar Sushruta (सुश्रुत). According to a twentieth century historian,^16^ its existence was first referenced (original source not available) in the sixth century BC. More recent studies assume a later initial conception between the second or first century BC with multiple contributors redacting passages.^17^ The 76 eye diseases with likely depictions of uveitis (*Adhi-mantha*) or glaucoma (*Gambhirika*) were probably added later in its volume *Uttara-Tantra.* Disease 54 (*Sannipatika Timira*) is suggestive of double images and oscillopsia caused by involuntary eye movements:

[…] the outer world looks variegated and confused, appears as doubled or trebled to the vision (of the patient), and stars and planets, either defective or supplied with additional limbs, seem to float about in the vision.

No specific treatment or prognosis is mentioned in the available texts. Contemporary alternative ayurvedic medicine offers a variety of herbal remedies for nystagmus such as *Triphala*, a mixture of fruits from Emblica officinalis (Amalaki), Terminalia bellerica (Bibhitaki) and Terminalia chebula (Haritaki) with antioxidative and anti-inflammatory properties.^18^

### Greece/Rome

The Hippocratic corpus^5^ is a collection of roughly 60 early ancient Greek medical works strongly associated with Hippocrates of Kos (Ἱπποκράτης ὁ Κῷος, approx.460 BC − 370 BC) and his teachings. It features a variety of ophthalmological diseases in the Book *About Vision*, including pterygium, traumatic injuries, amblyopia and pupil disorders.^19^ With only nine chapters preserved until the present day, large parts have been lost, including the chapters on ‘hippos’ caused by a ‘spasm of the eye’. These segments, attributed to Hippocrates, are found in later works attributed to Galenos of Pergamon^20^:

[…] it is similar to an eye disease . . . which is called hippos, in which it is not possible to keep the eyes still for a moment. . . they are forever oscillating back and forth with a trembling movement … they are unstable, much like those who have the so-called hippos and are not able to keep them quiet.

A direct quote from the Hippocratic corpus in the *prognosticon* gives this short description of ‘rapid pupil motion’:

εἰ δὲ καὶ σφυγμὸς ἐνείη ἐν τῷ ὑποχονδρίῳ, θόρυβον σημαίνει, ἢ παραφροσύνην: ἀλλὰ τοὺς ὀφθαλμοὺς ἐπικατιδεῖν τῶν τοιουτέων: ἢν γὰρ αἱ ὄψιες πυκνὰ κινέωνται, μανῆναι τούτους ἐλπίς.

[…] and the physician should examine the eyes of such persons; for if their pupils be in rapid motion, such persons may be expected to go mad.

Aulus Cornelius Celsus (approx. 25 BC – 50 AD) in *De Medicina*^21^ gives a detailed description of nystagmic oscillopsia:

Igitur interdum evenit, modo in altero oculo, modo in utroque, aut ex ictu aliquo aut ex morbo comitiali, aut ex distentione nervorum, qua vehementer ipse oculus concussus est, ut is neque quoquam intendi possit, neque omnino consistat, sed huc illucue sine ratione moveatur; ideoque ne conspectum quidem rerum praestat.

It happens then sometimes in the case of one eye, sometimes of both, from some blow, or from epilepsy, or from a spasm, by which the eyeball itself is violently shaken, that it cannot be directed at any object, or be held at all steady, but with no reason it turns now this way, now that, and so does not even afford a view of objects.

Aretaios of Cappadocia (Ἀρεταῖος, 80/81 AD – approx. 130–138 AD) describes in his book ‘*De causis et signis acutorum et chronicorum morborum*’ in the chapter (entitled ‘Περὶ κεφαλαίης’, ‘About Headache’) a hemicrania with vertigo, oscillatory eye movements (nystagmus?) and nausea that resembles our current definition of vestibular migraine^2^:

[…] ἑτεροκρανίη τόδε [μοῦνον] καλέεται …σπασμὸς καὶ διαστροφὴ τοῦ προσώπου γίγνεται ὀφϑαλμοὶ ἢ ἀτενέες κεράεσι ἴκελοι πεπήγασι, ἢ τῇδε κακεῖσε σπασμωδέως ἔνδον εἱλῶνται σκοτόδινος […]

[…]This type of headache is called heterocrania…The face is distorted spasmodically, the eyes remain glassy and rigid like horns or move to and fro forcedly, and the patient is dizzy […]^22^

Since the predominant pathophysiological theory of the Hippocratic school centered on the imbalance of blood, phlegm, yellow bile and black bile, therapeutic interventions usually involved attempts at rebalancing these fluids, for example, by phlebotomy.

### China

The Huangdi Neijing (黄帝内经^7^), the fundamental Chinese medical text book, dates back to between the 2^nd^ century BC and the 2^nd^ century AD. It was collected and eventually divided into its modern form of two volumes of 81 chapters or treatises each in 762 AD by Wang Bing, a high-ranking scholar in the Tang-Dynasty, while it was first mentioned centuries earlier in the Book of Han (111 AD).^23^ Several depictions of abnormal eye movements can be found like this text in verse:

[People suffer from] fright, spasms, coughing, and nosebleed;

their hearts are hot, vexed, and overexcited.

They frequently relieve themselves and they dislike wind.

receding qi moves upward.

The face looks as if covered by dust.

The eyes have twitching and spasms.

fire qi is effused internally and causes oral putrescence because of vomiting and [qi] moving contrary [to its

regular course].

[Patients suffer from] blood overflow and blood outflow.^24^

In severe cases, [patients hear] a ringing [sound] in the ears and [they experience] dizziness

and vertigo

Their eyes fail to recognize other persons.

They tend to suddenly fall.^24^

The following passage has been interpreted as recalling a typical attack of Menière's disease^2^:

If Qi is insufficient above, the brain is not sufficiently filled by it, the ears suffer a ringing noise, the head is bent low by it, the eyes [experience] dizziness.

No eye-movement-specific therapy is proposed in the Huangdi Neijing. Since diseases were thought to involve inadequate supply of specific body substances, such as blood and the vital force Qi, rebalancing of one's Qi or other body fluids were suggested as general remedies.

### Middle East

Hunain ibn Is-hâq (أبو زيد حنين بن إسحاق لعبادي, 808–873 AD) was a Christian-Arab scholar who wrote a systematic textbook of ophthalmology and translated works by Galenos from Greek and Aramaic primary sources that are now lost.^25^ In his scripts, he describes ophthalmoplegia and eye movements as ‘paralysis’ and ‘spasms’ of the eye muscles, respectively.

The lesions happening to the voluntary motion of the eye are of three kinds: in the first the motion ceases; this is called paralysis, laming; in the second it is diminished, and this is called numbness and trembling; in the third the voluntary motion is a disturbed one, i.e. other than it is the intention of the moving agent to produce, and this is called spasm.

No information on therapeutics at that time was discernible.

## Discussion

Potential short descriptions of pathological involuntary eye movements are preserved in many ancient documents such as papyrus scrolls, stone tablets or books (Figure 1). Medical categorisation, however, according to our current classification of eye movement disorders, remains vague and speculative, partially due to inconsistent translations or the retrospectively confusing development of terminology or the fragmentary nature of the sources. As stated by Karenberg,^26^ retrospective diagnoses require a careful interpretation of ancient sources in the context of the texts. Hypothesis-driven far-reaching assignments to specific forms of nystagmus are judged critically by historically experienced neuro-ophthalmologists.^27^ On the other hand, there are examples of comprehensible and convincing descriptions of certain clinical syndromes and vestibular disorders based on the combination of signs and symptoms. One example is the above-described syndrome of hippos by Hippocrates and Galen as a congenital condition which allows two clinical attributions, either a congenital (infantile) nystagmus or the continuous involuntary eye movements of the blind. Further, a Greek text by Aretaios is compatible with episodic attacks of vestibular migraine.^2^ In the current study, we even found a historically earlier reference to a combination of hemicrania with involuntary ocular oscillations on cuneiform stone tablets dating back to the 7^th^ century BC in Mesopotamia. The Chinese textbook Huangdi Neijing contains passages which persuasively suggest attacks of Menière's disease.^2^ Extensive text sources from Greek, Roman and Chinese antiquity can be found on other vertigo syndromes such as motion sickness/seasickness^28,29^ and height vertigo/acrophobia,^30,31^ however, without explicit mention of associated eye movements.

In the current study, the ancient attempts to relate pathological eye movements to underlying diseases and pathophysiologies are, in some cases, plausible from today's point of view. They contain fever in neuroinfections, circulatory disorders, epilepsy or head trauma. Our translation of the original text of Celsus from Latin^21^ into English also includes the visual consequences of acquired involuntary ocular oscillations, which obviously refer to oscillopsia. While the assumed underlying pathomechanism depends on cultural context and often involves mythical beliefs, therapeutic approaches were limited to topical ointments or iatromagical ceremonies.

Contemporary clinical assessment of nystagmus syndromes involves a variety of aspects such as nystagmus type (e.g. pendular nystagmus, jerk nystagmus) and beating direction/frequency. Further aspects include binocularity, dissociation (e.g. dissociated nystagmus in internuclear ophthalmoplegia or in muscular paresis), effects of vergence, visual fixation or positioning manoeuvres as well as accompanying symptoms. A detailed description of nystagmus features is crucial in the diagnosis of the underlying pathology and often allows for exact lesion localisation. For some of these characteristics, possible equivalent descriptions can be inferred from ancient texts, such as Celsus’ differentiation of monocular and binocular presentation of eye movements or the separate terminology for vertical and torsional eye movements found on Mesopotamian stone tablets.

The historical processing of the different preserved ancient observations critically depends on the etymological development of the clinical sign of ‘nystagmus’. The modern nomenclature was first noted in 1763, where François Boissier de la Croix de Sauvages mentioned ‘nystagmus bulbi’ as synonymous with Galen's ‘hippos’, differentiating it from the now introduced ‘hippus’ as related to pupillary movements.^32^ The latter means spasmodic rhythmic dilating and contracting pupillary movements by the sphincter and dilatator muscles. This terminology was first used by Burchard Mauchart (1696–1751AD), a South-German pioneer of ophthalmology.^33^ The term ‘nystagmus’ (derived from νυσταγμός, ‘drowsiness, falling asleep’, inspired by the nodding movements of the head when in a drowsy state with short-term sleep phases associated with rhythmic loss of neck muscle tone) was introduced in the eighteenth century.

## Conclusion

Descriptions of nystagmus or other forms of abnormal involuntary eye movements, associated symptoms (e.g. vertigo, seizures, fever) and their impact on patients’ quality of life (e.g. oscillopsia, blurred vision) are preserved in fragments in numerous ancient documents such as papyrus scrolls and stone tablets originating from Egypt, Mesopotamia, India, China, Greece, Rome and the Middle East. These sources span from the 2^nd^ millennium BC to the ninth century AD. Ocular oscillations termed ‘hippos’ by Hippocrates (460–370BC) and Galenos (129–216AD) are synonymous with nystagmus, a term first introduced in the eighteenth century. Treatment options in antiquity ranged from religious ceremonies to topical ointments. Due to the limited medical accuracy of the sources, caution is required when attempting to categorise ancient descriptions of abnormal eye movements into distinct nystagmus forms according to our current clinical classification of ocular motor disorders.

## References

[1] Leigh RJ Zee DS . The neurology of eye movements*.* 5th ed. Oxford: Oxford University Press, 2015.

[2] Huppert D Brandt T . Descriptions of vestibular migraine and Menière's disease in Greek and Chinese antiquity. Cephalalgia. 2017; 37: 385–390. 27129480 10.1177/0333102416646755

[3] Wade NJ Tatler BW . The moving tablet of the eye: The origins of modern eye movement research. Oxford: Oxford University Press, 2010.

[4] Brandt T . A technical eye inspired by biology. Brain. 2006; 129: 1070–1073.

[5] Hippocrates, Francis A. The genuine works of Hippocrates. William Wood & Co., 1886.

[6] Thompson HS Franceschetti AT Thompson PM . Hippus. Am J Ophthalmol. 1971; 71: 1116–1120. 4935041

[7] Wang H . Huangdi neijing yanjiu dacheng: The great compendium of the research on the Huangdi Neijing. Beijing: Beijing chubanshe, 1997.

[8] Suśruta सK . An English translation of the Sushruta samhita: based on original Sanskrit text, with a full and comprehensive introduction, additional texts, different readings, notes, comparative views, index, glossary and plates. Varanasi: Chowkhamba Sanskrit Series Office, 1963.

[9] Breasted JH . The Edwin Smith papyrus. New-York Historical Society, 1922.

[10] Chapman PH . Case seven of the Smith surgical Papyrus: the meaning of TPȝW. J Am Res Center Egypt. 1992; 29: 35.

[11] Fincke JC . Augenleiden nach keilschriftlichen Quellen: Untersuchungen zur altorientalischen Medizin. Würzburg: Königshausen & Neumann, 2000.

[12] Gurney OR . The Sultantepe Tablets. By O. R. Gurney and J[acob] J[oel] Finkelstein (2: O. R. Gurney and P. Hulin). 1.2. 1957.

[13] Scurlock JA Andersen B . Diagnoses in Assyrian and Babylonian Medicine : Ancient sources, translations, and modern medical analyses. Baltimore, United States: University of Illinois Press, 2005.

[14] CDLI - Cuneiform Digital Library Initiative. 2022. https://cdli.ucla.edu/. Accessed August 1, 2022.

[15] Geller MJ . Mesopotamian eye disease texts: The nineveh treatise. Boston: Walter de Gruyter GmbH, 2020.

[16] Hoernle AFR . Studies in the medicine of ancient India. Part I. Osteology, or The bones of the human body. Oxford: Clarendon Press, 1907.

[17] Meulenbeld GJ . A history of Indian medical literature. Groningen: Forsten, 1999.

[18] Peterson CT Denniston K Chopra D . Therapeutic uses of Triphala in ayurvedic medicine. J Altern Complement Med 2017; 23: 607–614. 28696777 10.1089/acm.2017.0083 PMC5567597

[19] Lascaratos J Marketos S . Ophthalmological lore in the Corpus Hippocraticum. Doc Ophthalmol 1988; 68: 35–45. doi:10.1007/BF00153586. 3046869

[20] Kühn KG (ed.) Claudii Galeni Opera Omnia: Volume 8. Cambridge University Press, 1821.

[21] Celsus AC . [Books I - IV]. Cambridge, Mass.: Harvard University Press, 1971.

[22] Koehler PJ van de Wiel TW . Aretaeus on migraine and headache. J Hist Neurosci 2001; 10: 253–261. 11770192 10.1076/jhin.10.3.253.9089

[23] Knechtges DR Chang T (eds) Ancient and early medieval Chinese literature: A reference guide, volume 1. Leiden, Boston: Brill, 2010.

[24] Unschuld PU . Huang Di nei jing su wen: Nature, knowledge, imagery in an ancient Chinese medical text, with an appendix, the doctrine of the five periods and six qi in the Huang Di nei jing su wen. Berkeley: University of California Press, 2003.

[25] Hunayn ibn Ishaq al-`Ibadi, Meyerhof, Max. The Book of the ten treatises on the eye ascribed to Hunain ibn Is-haq: the earliest existing systematic text-book of ophthalmology. Cairo: Government press, 1928.

[26] Karenberg A . Retrospective diagnosis: use and abuse in medical historiography. Prague Med Rep [serial online]. 2009; 110: 140–145. 19591388

[27] Böck B . Diagnose im Alten Mesopotamien. Überlegungen zu Grenzen und Möglichkeiten der Interpretation keilschriftlicher diagnostischer Texte. Orientalistische Literaturzeitung 2009; 104: 381–398.

[28] Brandt T Bauer M Benson J , et al. Motion sickness in ancient China: seasickness and cart-sickness. Neurology 2016; 87: 331–335. 27432177 10.1212/WNL.0000000000002871

[29] Huppert D Oldelehr H Krammling B , et al. What the ancient Greeks and Romans knew (and did not know) about seasickness. Neurology 2016; 86: 560–565. 26857952 10.1212/WNL.0000000000002355

[30] Huppert D Wuehr M Brandt T . Acrophobia and visual height intolerance: advances in epidemiology and mechanisms. J Neurol 2020; 267: 231–240. 32444982 10.1007/s00415-020-09805-4 PMC7718183

[31] Bauer M Huppert D Brandt T . Fear of heights in ancient China. J Neurol 2012; 259: 2223–2225. 22584951 10.1007/s00415-012-6523-5

[32] La Croix Sauvages FB de . Nosologia methodica sistens morborum classes, genera et species, juxta Sydenhami mentem et botanicorum ordinem. Fratrum de Tournes, 1763.

[33] Hirschberg J . Geschichte der Augenheilkunde*.* 1899th ed. Norderstedt Hansebooks GmbH, 2017.

